# The Nordic back pain subpopulation program - individual patterns of low back pain established by means of text messaging: a longitudinal pilot study

**DOI:** 10.1186/1746-1340-17-11

**Published:** 2009-11-17

**Authors:** Alice Kongsted, Charlotte Leboeuf-Yde

**Affiliations:** 1 Nordic Institute of Chiropractic and Clinical Biomechanics, Clinical Locomotion Science, Forskerparken 10A, 5230 Odense M, Denmark; 2 Research Unit for Clinical Biomechanics, University of Southern Denmark, Campusvej 55, 5230 Odense M, Denmark; 3 The Back Research Center, Clinical Locomotion Science, Lindevej 5, 5750 Ringe, Denmark

## Abstract

**Background:**

Non-specific low back pain (LBP) is known to be a fluctuating condition and there is a growing realisation that it consists of different subgroups of patients. The detailed course of pain is not known since traditional methods of data collection do not allow very frequent follow-ups. This is a limitation in relation to identification of subgroups with different course patterns. The objective of this pilot study was to see if it is possible to identify characteristic course-patterns of non-specific LBP in patients treated in a primary care setting.

**Methods:**

Patients seeing a chiropractor for a new LBP episode were included after the first consultation and followed for 18 weeks by means of automatic short message service (SMS) received and returned on their mobile phones. Every week they were asked how many days they had experienced LBP in the preceding week. The course of pain was studied for each individual and described as an early course (1^st ^- 4^th ^week) and a late course (5^th ^- 18^th ^week), which was fitted into one of 13 predefined course patterns.

**Results:**

A total of 110 patients were included from 5 chiropractic clinics, and the study sample consisted of the 78 patients who participated at least until week 12. Nine of the predefined patterns were identified within this population. The majority of patients improved within the first four weeks (63%), and such early improvement was associated with a generally favourable course.

**Conclusion:**

Patients with nonspecific LBP were shown to have a number of different course-patterns. The next step is to explore whether the identified patterns relate to different LBP diagnoses.

## Background

Despite numerous studies into risk factors for developing [1-4] or not recovering [5] from non-specific low back pain (LBP) there has been no real breakthrough in relation to its causes. Without knowledge of the causes it is not surprising that the various treatment approaches have also failed to produce any outstanding results [6-10]. One reason for this lack of progress might be that the traditional methodological approach is to consider LBP as a condition with a well-defined course, resembling that of many other diseases with an acute course which for some people may develop into a sub-acute or even a chronic stage. However, it has more recently become apparent that LBP is an episodic or fluctuating condition for many patients [11-15], meaning that we do not really know when the problem starts and when it ends. This fact needs to be taken into account when studying causes and treatment effects.

The fluctuating nature of LBP has been established through population-based surveys [15], in LBP patients responding to a newspaper advertisement [16], by studying recovery patterns in primary care patients [17,18], and in workers who have been sick-listed because of back pain [19,20].

Interestingly, different patterns of recovery have been identified through interviews at up to five points in time [20] and through monthly surveys [17] over a one-year period. Such patterns went from large improvement through fluctuating symptoms to continuous pain. In addition, even relatively substantial daily fluctuations have been observed [16]. It seems reasonable that such different course patterns also have different causes and perhaps require different treatment approaches too, and they therefore merit a closer scrutiny.

It has been suggested previously that the identification of distinct subgroups of LBP patients is a necessary step in order to advance our understanding of the causes of LBP and thereby also the indications for treatment as well [21-25]. However, it is difficult to identify subgroups when neither cause nor indications for suitable treatments are well understood. Perhaps the different course patterns that LBP exhibits over time could help identify homogenous subgroups that would have a more clear-cut response to treatment. Moreover it is possible that different course patterns relate to different pain generators. E.g. that a relatively fast recovery is expectable from pain due to simple mechanical dysfunction, whereas it is likely that discogenic pain will improve slower and be fluctuating for some time after the initial improvement.

To be able to identify characteristic course patterns of LBP in more detail it will be necessary to follow persons with LBP frequently and regularly over a long period of time. Spot checks at long intervals using questionnaires or telephone interviews are not suitable because they depend on study subjects having a very reliable long-term memory, and these methods are not feasible for frequent follow-ups. A better method is to use daily pain diaries, providing of course that these are not filled out retrospectively, when it is time to return the diaries. However, both diaries and repeated questionnaires require a fair amount of co-operation from the study subjects and are also relatively expensive.

In order to overcome some of these methodological short-comings and to be able to observe the fluctuations in pain patterns, short message service (SMS) that is automatically sent to respondents' mobile phones has been introduced as a tool for frequent surveillance. By sending standardized questions by text messages to participants and incorporating their replies directly into a data file, it is possible to perform frequent data collection in a cheap and easy way.

The study was conducted as a pilot study and the main objective was to see if it is possible to identify characteristic course-patterns of LBP in patients treated in a primary care setting when collecting self-reported data once a week. Information regarding the number of LBP days the previous week, number of days off work due to LBP, and pain intensity present on the day of the follow-up was gathered over a period of 18 weeks. A secondary objective of the study was to learn about the SMS method both in relation to data collection and in relation to the data analysis.

## Methods

### Study design

This was a multi-centre longitudinal observational study.

### Participants

Patients were recruited by chiropractors in private clinics. Inclusion criteria were: LBP with or without sciatica as the main complaint, 18 - 65 years old and having a mobile phone. Patients were not included if one of the following non-inclusion criteria were present: Previous back surgery, pregnancy, other significant musculoskeletal problems in addition to the LBP, inability to read or speak Danish. Prior to inclusion patients received written and verbal information about the study. The project was presented for the local ethics committee which found that it did not need approval.

### Clinical procedures

Patients who agreed to participate had a standardised clinical examination and were assigned a mechanical diagnosis based upon this [26]. Chiropractors were free to choose whichever type and frequency of treatment they found appropriate and registered what treatment they had initiated.

### Follow-up procedure

Follow-up was conducted by means of SMS. Text messages were automatically sent to the participants' mobile phones starting on the first Sunday following inclusion and thereafter repeated every Sunday for 18 weeks. One SMS was sent for each of three follow-up questions, and replies were given by answering each SMS directly on the phone. If the SMS had not been answered on the first-coming Thursday, a reminder was automatically sent. The text message information returned by study participants was automatically incorporated into a data file hosted on a server at the provider of the SMS-track system's office [27].

### SMS questions

Each week participants received three questions to which they sent their answers one by one:

Question 1. Please answer how much your lower back hurts today? Choose a number: 0 = no pain at all/1 = some pain/2 = severe pain

Question 2. Using a number from 0 to 7, please answer how many days you have been bothered by your lower back this week.

Question 3. Using a number from 0 to 7, please answer how many days you have been off work because of your lower back this week. (Answer with X if you are not working)

In a previous report on the same study, it was noted that there were virtually no differences between the patterns for number of days with LBP (questions 2) and severity of pain (question 1) on a group level. Moreover, days off work (question 3) were too infrequent to be suitable for the analysis of course profiles. For these reasons, this report deals only with the number of days that subjects were bothered by their lower back, as reported on a weekly basis.

### Data analysis

Data were transmitted from a spread sheet to STATA 10.1 (StataCorp, Texas, USA). When answers other than a number were given, data was recoded as a number when possible, e.g. "I have no pain" was recoded as 0, and "2 days last week" as 2. Answers that could not be transformed directly into a number were coded as missing values. The final study sample consisted of patients who participated at least until the 12^th ^week with no more than two weeks pause in a row.

A plot showing number of days bothered from the lower back (LBP-days) for each week was created for each participant. Based upon knowledge about the importance of the early course of LBP [28-30], it was decided to describe the pain course in two stages: The early course (week 1 - 4) and the later course (week 5 - 18). A previous population-based analysis indicated that the main changes had taken place at the 4^th ^week, justifying this as a suitable cut-point also in this study.

It was hypothesized that the courses could be towards improvement or worsening, could be fluctuating or that no changes would occur. On the basis of this assumption, a visual model for categorising the individual pain courses was defined. The model was tested by the authors separately on 20 curves, after which the model was adjusted until it was possible to fit all curves. The final visual model divided the early course into three groups ("improved", "unchanged" and "worsened"). The later course was described as "mainly recovered", "stays in the initial category", "moves towards mainly improved", "fluctuates", or "moves towards mainly worsened". In total, this resulted in 15 theoretically possible categories. Two of those were left out because the categories "improves and then moves towards mainly improved" and "worsened and then moves towards mainly worsened" would instead appear in those described as "stays in the initial category" (Table 1 and Table 2).

**Table 1:** Definitions of the categories used to describe the identified course patterns

Categories used to describe the early course (weeks 1 - 4)	Definitions
Improved	≥ 2 days reduction in LBP-days/week when comparing week 4 to week 1

Unchanged	The same number of LBP-days +/- 1 day when comparing week 4 to week 1

Worsened	≥ 2 days more with LBP-days/week when comparing week 4 to week 1

**Categories used to describe the late course** **(weeks 5 - 18)**	

Mainly recovered	A maximum of one week with any LBP-days during the late course

Stays in the initial category	The number of LBP-days stays within the limits of the category that was assigned in the early course

Moves towards mainly improved	Moves from unchanged or worse in the early course to being improved (reporting ≥ 2 days reduction in LBP-days/week when compared to week 1), and has a maximum of one week outside that category *(The category does not apply to those who were improved in the early course)*

Fluctuating	Fluctuates between improved, unchanged, or worse as compared to week one

Moves towards mainly worsened	Moves from improved or unchanged in the early course to being worse (reporting ≥ 2 days more with LBP-days/week than in week 1), and has a maximum of one week outside that category *(The category does not apply to those who were worsened in the early course)*

**Table 2:** Possible combinations of early and late course into final categories

At the 4^th ^week	Late courses which the early course can possibly be combined with
Improved	Mainly recovered Stays in the category Fluctuating Moves towards mainly worsened

Unchanged	Mainly recovered Stays in the category Moves towards mainly improved Fluctuating Moves towards mainly worsened

Worsened	Mainly recovered Stays in the category Moves towards mainly improved Fluctuating

The possible combinations that form thirteen categories, which describe pain courses during 18 weeks. First the appropriate category "at the 4th week" was chosen and afterwards the subsequent course.

When the model had been finalized, both authors allocated all participants into one of the 13 categories. This resulted in agreement regarding 62/78 = 79.5% of the curves (kappa 0.74; 95% CI 0.68 - 0.78), which is interpret as a substantial agreement [31]. The curves that had been categorized differently by the two authors were re-evaluated and consensus was obtained regarding their allocation. Information on the course patterns in relation to past history, duration of symptoms and the different diagnostic subgroups will be reported elsewhere.

## Results

### Participants and response rates

Seven female chiropractors with an average of 7.6 years of clinical experience from five chiropractic clinics in Denmark included patients for the study. Six chiropractors had graduated from the University of Southern Denmark and one from the Palmer College, California, USA.

**Figure 1:**
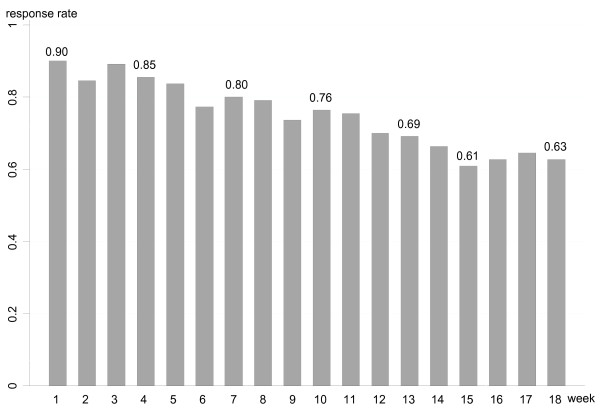
**The percentage of LBP patients who responded to SMS-questions for each week among those who accepted inclusion at the first visit to a chiropractor**.

One hundred and ten patients agreed to participate and 101 responded to the first text message. The follow-up rate declined as the study period went on with 86%, 78% and 70% of the participants who answered in week one still responding in weeks 6, 12 and 18 respectively (Fig. 1). A comparison between responders and non-responders revealed that those who dropped out were more likely to be men, present to the chiropractor with acute LBP, and have leg pain in addition to LBP.

**Figure 2:**
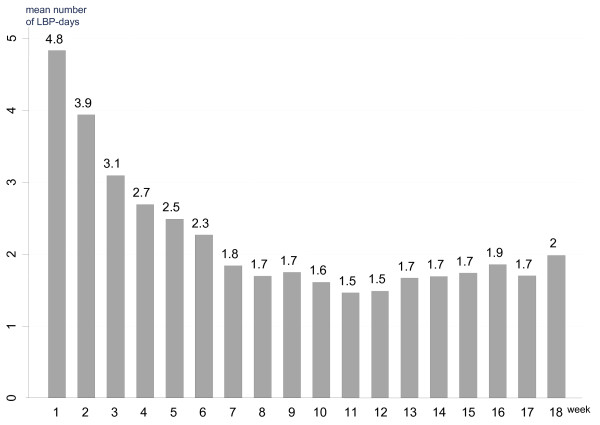
**The mean number of reported LBP-days following the initial visit to a chiropractor for each week during an 18-week study**.

The final study sample for the identification of pain patterns consisted of 78 patients (39 men and 39 women, mean age 42.5 years (SD 9.9)). Other characteristics of the study population and the type of treatment initiated at the first visit appear from Table 3. Several treatment modalities were often used in combination. On a group level, the highest mean number of LBP-days was observed in week one and the lowest number in week 11 and week 12 (Fig. 2).

**Table 3:** Duration of pain at baseline, type of treatment, and response to the first treatment

Participants	n = 78
**LBP at baseline**	
acute (1 - 7 days) sub-acute (8 days - 3 months)	45% 33%
chronic (> 3 months)	19%
missing	3%

**Type of treatment**	
manipulation	85%
mobilization	14%
soft tissue technique	60%
information/advice	82%
exercise (any type)	32%

**Status after 1^st ^treatment**	
much better	12%
better	36%
unchanged	33%
worse	12%
much worse	5%
missing	3%

Duration of pain and type of treatment registered at the first visit to a chiropractor. Self-reported status after 1st treatment was collected by means of automatic test messages.

### Are there characteristic course patterns?

#### Description of the early individual course patterns

Of the 78 participants 63% were categorized as improved, 30% as unchanged and 6% as worsened at the end of the 4^th ^week (last column Table 4). Data were missing from one participant who did not answer during the first two weeks.

**Table 4:** Percentage distribution of the defined course patterns in 78 patients with LBP [n (%)]

			5^th ^to 18^th ^week			
**At the 4th week**	**Mainly recovered**	**stays in the initial category**	**moves - towards mainly improved**	**Fluctuating**	**moves - towards mainly worse**	**total**

Improved	11 (14%)	31 (40%)	NA	7 (9%)	0	49 (63%)

Unchanged	3 (4%)	2 (3%)	6 (8%)	12 (15%)	0	23 (30%)

Worsened	0	1 (1%)	0	4 (5%)	NA	5 (6%)

(missing)	1 (1%)	0	0	0	NA	1 (1%)

	15 (19%)	34 (44%)	6 (8%)	23 (29%)	0	78 (100%)

Refer to Table 1 for definitions of the categories. NA = non-applicable.

#### Description of the late individual course patterns

During the rest of the study period (weeks 5 to 18) 19% of the 78 patients were categorized as mainly recovered, 44% remained in their initial category (improved, unchanged or worsened), 29% moved between categories, and 8% moved from the initial category towards mainly improved, while none moved from improved or unchanged in the early course towards mainly worse (bottom row in Table 4). The subject, whose early course was missing, recovered in the late course.

#### Description of the total individual course pattern

**Figure 3:**
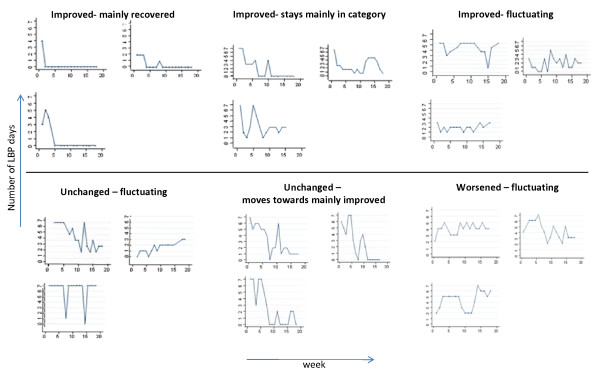
**Examples of individual LBP courses within the categories holding at least 5% of participants**.

Nine of the 13 possible course patterns were observed in this study sample (Table 4). Examples of the way the course patterns were plotted appear from Fig. 3. Early improvement that was followed by either full recovery or staying somewhat improved was found in 54% of the population.

The most frequent pattern was early improvement and staying improved as compared to week one but not being totally recovered (40%) Also, the "fluctuating" patterns with patients moving between categories in the late course were frequent (29%).

Four of the hypothesised categories were found to be non-existing: Patients with early worsening did not recover or move to mainly improved; patients who were improved after the 4^th ^week did not move to mainly worse; and patients who were unchanged after 4 weeks did not move to mainly worse.

The 23 patients who were unchanged after the 4^th ^week appeared to have rather unpredictable courses, although none moved to the "mainly worse" category. The five patients who got worse in the early course were most likely to have a fluctuating pattern, and only one patient was classified as getting worse and staying in this category.

#### Number of LBP days related to the main patterns

**Figure 4:**
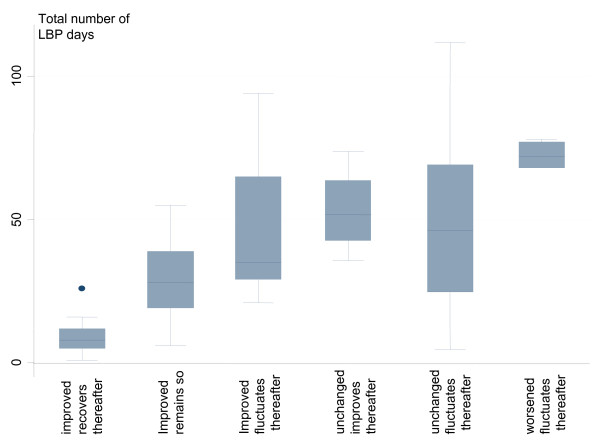
**Number of LBP-days during the entire 18-weeks study within each defined course pattern**.

The mean total number of LBP-days during the entire follow-up period was 36.2 (SD 28) for the entire population. The total number of LBP days for each course profile is illustrated in Fig. 4. Patients who had early improvement and recovered in the late course had the lowest number of LBP days, followed by patients who were unchanged in the early course to recover in the later period. There was a larger variation within the groups with fluctuating patterns than within the groups that moved to mainly improved or who recovered. Patients who had a LBP pattern of worsening in the early course had the highest number of LBP days in total.

## Discussion

To our knowledge, this study was the first to describe individual pain profiles in LBP based upon weekly follow-ups. We found that distinct patterns exist within non-specific LBP, and that such patterns can be identified even in a small study sample. Obviously, our results do not describe the natural course of LBP since participants received different kinds of manual care during the study period. Instead, our results are probably typical for people who have decided to seek care for their LBP in the primary care sector. Because it was a pilot study with a small study sample any uncommon pain patterns would not have been captured and identified. This would require larger study samples and different settings.

Prior to the data analysis we assumed that thirteen different pain patterns would exist. Nine of these patterns were represented and six of these were fairly frequent. Two extreme groups were identified. One consisted of patients who improved quickly and remained recovered. Not surprisingly, this group had the fewest number of days with LBP in total over the 18 weeks study period. The second group consisted of those who worsened in the early course and thereafter had a fluctuating course. This group had the largest total number of days with LBP in the study period. The least common pattern was early deterioration followed by an unfavorable long-term development. The most common pattern was early improvement followed by a good or a relatively good long-term development.

Most clinicians treating patients with LBP in a primary care setting probably strive for a relatively stable recovery. However, in this study only 19% could be classified as recovered. In fact, our results suggest that clinicians instead should expect that patients exhibit a rather fluctuating course.

In addition this pilot study was designed to find out if the method of collecting data with automatic text messages (SMS-Track) was useful in detecting different course patterns. It seems like weekly monitoring can reveal fluctuations in the course of LBP that might be missed using follow-ups at longer intervals. This perhaps explains why Dunn et al[17], who studied this subject using monthly questionnaires, noted that 13% of patients with LBP in the primary care sector had a fluctuating pattern whereas our estimate was about twice as high. It would, however, be necessary to study this phenomenon also in other study populations. It has been suggested that LBP status should be measured through number of days in pain over a period rather than counting those still in pain at the end of the period [32]. The SMS-method, as described in our study, would be suitable for this purpose.

Although this method has the obvious strength of being able to collect data while the study subjects still remember the answers, it also has some weaknesses. For example, only few and short questions are suitable for text-messaging and participants must have a mobile phone and know how to use text messaging. On the other hand, our experience with the practical aspects of this method was favorable. The direct incorporation of the respondents' answers into a data file meant that there was no manual entry of data. Hence, human resources were economized and we avoided a potential source of error.

In the present study the main weakness was a poor response rate with only 63% of the participants responding at the last follow-up after 18 weeks. This is in line with the response rate obtained by mailed questionnaires after 3 months in another chiropractor patient population [13], but not as high as a 93% response rate achieved using telephone interviews of chiropractor patients [28]. However, our response rate might be as large as can be expected with frequent follow-ups. In a study of LBP patients in general practice only 44% responded to at least 4 out of 6 monthly questionnaires [17]. We believe our lower response rate resulted from participant fatigue due to the frequency of follow-ups, but we also find it very likely that it is possible to improve response rates in future studies of this type. It would take an increased effort put into informing patients about the study prior to inclusion, and that non-responders are contacted verbally on the phone when they first miss a follow-up in order to clarify any misunderstandings. Since our non-responders had a longer duration of pain prior to inclusion than responders, and more often had leg pain in addition to back pain, the low response rate may have resulted in a too optimistic picture of the LBP-course within this population.

We chose a pragmatic way of analyzing the data. The classification of the curves used to illustrate the various course patterns was performed manually through simple inspection of the printed curves. A clear-cut definition was made for each parameter used for the classification and the agreement on the classification between the two authors was substantial. This approach made it possible to identify clinically meaningful patterns, but would not be feasible when analyzing data from larger cohorts. Therefore, other methods of analysis will be described elsewhere.

Our next step will be to verify if our subgroups should be altered, i.e. further subdivided, or if some of the groups actually are so alike that they should be collapsed into to fewer groups. We also intend to explore whether the identified patterns relate to specific LBP diagnoses, and if they can be predicted from baseline characteristics. If so, we would come closer to the identification of clinically relevant subgroups in LBP.

## Conclusion

In conclusion, our preliminary results are promising in that we could identify several distinct groups of patients with different LBP course patterns. We tested a new method to collect data and found it to be easily used, although more effort should be placed on informing the patient about the requirements of the study to minimize loss to follow-up.

Our findings indicate that most patients with LBP who seek chiropractic care improve in the early course, i.e. within the first four weeks, and that such early improvement was often associated with a generally good course. However, even among patients with early improvement the majority do not experience a full recovery. An important finding was that a fluctuating course is relatively common among these patients. Obviously, this should be taken into account from both clinical and research perspectives.

## Competing interests

The authors declare that they have no competing interests.

## Authors' contributions

Both authors participated in the design of the study, data analysis and drafting of the manuscript. AK instructed the chiropractors who included patients and collected the data.

## References

[B1] Rubin DI Epidemiology and risk factors for spine pain Neurol Clin 2007 17 353 371 10.1016/j.ncl.2007.01.004 17445733

[B2] Hartvigsen J Leboeuf-Yde C Lings S Corder EH Is sitting-while-at-work associated with low back pain? A systematic, critical literature review Scand J Public Health 2000 17 230 239 11045756

[B3] Leboeuf-Yde C Body weight and low back pain. A systematic literature review of 56 journal articles reporting on 65 epidemiologic studies Spine 2000 17 226 237 10.1097/00007632-200001150-00015 10685488

[B4] Leboeuf-Yde C Smoking and low back pain. A systematic literature review of 41 journal articles reporting 47 epidemiologic studies Spine 1999 17 1463 1470 10.1097/00007632-199907150-00012 10423792

[B5] Kent PM Keating JL Can we predict poor recovery from recent-onset nonspecific low back pain? A systematic review Man Ther 2008 17 12 28 10.1016/j.math.2007.05.009 17658288

[B6] Hagen KB Jamtvedt G Hilde G Winnem MF The updated Cochrane review of bed rest for low back pain and sciatica Spine 2005 17 542 546 10.1097/01.brs.0000154625.02586.95 15738787

[B7] Furlan AD van TM Cherkin D Tsukayama H Lao L Koes B Berman B Acupuncture and dry-needling for low back pain: an updated systematic review within the framework of the Cochrane collaboration Spine 2005 17 944 963 10.1097/01.brs.0000158941.21571.01 15834340

[B8] Furlan AD Imamura M Dryden T Irvin E Massage for low back pain: an updated systematic review within the framework of the Cochrane Back Review Group Spine (Phila Pa 1976) 2009 17 1669 1684 19561560 10.1097/BRS.0b013e3181ad7bd6

[B9] Assendelft WJ Morton SC Yu EI Suttorp MJ Shekelle PG Spinal manipulative therapy for low back pain Cochrane Database Syst Rev 2004 CD000447 14973958 10.1002/14651858.CD000447.pub2

[B10] Hayden JA van Tulder MW Malmivaara A Koes BW Exercise therapy for treatment of non-specific low back pain Cochrane Database Syst Rev 2005 CD000335 16034851 10.1002/14651858.CD000335.pub2 PMC10068907

[B11] Wasiak R Young AE Dunn KM Cote P Gross DP Heymans MW Von KM Back pain recurrence: an evaluation of existing indicators and direction for future research Spine 2009 17 970 977 10.1097/BRS.0b013e3181a01b63 19532005

[B12] Von Korff M Saunders K The course of back pain in primary care Spine 1996 17 2833 2837 10.1097/00007632-199612150-00004 9112707

[B13] Leboeuf-Yde C Grønstvedt A Borge JA Lothe J Magnesen E Nilsson O Røsok G Stig LC Larsen K The Nordic back pain subpopulation program: a 1-year prospective multicenter study of outcomes of persistent low-back pain in chiropractic patients J Manipulative Physiol Ther 2005 17 90 96 10.1016/j.jmpt.2005.01.010 15800507

[B14] Croft PR Macfarlane GJ Papageorgiou AC Thomas E Silman AJ Outcome of low back pain in general practice: a prospective study BMJ 1998 17 1356 1359 9563990 10.1136/bmj.316.7141.1356 PMC28536

[B15] Hestbaek L Leboeuf-Yde C Engberg M Lauritzen T Bruun NH Manniche C The course of low back pain in a general population. Results from a 5-year prospective study J Manipulative Physiol Ther 2003 17 213 219 10.1016/S0161-4754(03)00006-X 12750654

[B16] McGorry RW Webster BS Snook SH Hsiang SM The relation between pain intensity, disability, and the episodic nature of chronic and recurrent low back pain Spine 2000 17 834 841 10.1097/00007632-200004010-00012 10751295

[B17] Dunn KM Jordan K Croft PR Characterizing the course of low back pain: a latent class analysis Am J Epidemiol 2006 17 754 761 10.1093/aje/kwj100 16495468

[B18] Stanton TR Henschke N Maher CG Refshauge KM Latimer J McAuley JH After an episode of acute low back pain, recurrence is unpredictable and not as common as previously thought Spine 2008 17 2923 2928 10.1097/BRS.0b013e31818a3167 19092626

[B19] Marras WS Ferguson SA Burr D Schabo P Maronitis A Low back pain recurrence in occupational environments Spine 2007 17 2387 2397 10.1097/BRS.0b013e3181557be9 17906584

[B20] Chen C Hogg-Johnson S Smith P The recovery patterns of back pain among workers with compensated occupational back injuries Occup Environ Med 2007 17 534 540 10.1136/oem.2006.029215 17387134 PMC2078491

[B21] Leboeuf-Yde C Manniche C Low Back Pain: Time to get off the treadmill J Manipulative Physiol Ther 2001 17 63 66 10.1067/mmt.2001.112009 11174697

[B22] Childs JD Fritz JM Flynn TW Irrgang JJ Johnson KK Majkowski GR Delitto A A clinical prediction rule to identify patients with low back pain most likely to benefit from spinal manipulation: a validation study Ann Intern Med 2004 17 920 928 15611489 10.7326/0003-4819-141-12-200412210-00008

[B23] Long A Donelson R Fung T Does it matter which exercise? A randomized control trial of exercise for low back pain Spine 2004 17 2593 2602 10.1097/01.brs.0000146464.23007.2a 15564907

[B24] Brennan GP Fritz JM Hunter SJ Thackeray A Delitto A Erhard RE Identifying subgroups of patients with acute/subacute "nonspecific" low back pain: results of a randomized clinical trial Spine 2006 17 623 631 10.1097/01.brs.0000202807.72292.a8 16540864

[B25] Fritz JM Brennan GP Clifford SN Hunter SJ Thackeray A An examination of the reliability of a classification algorithm for subgrouping patients with low back pain Spine 2006 17 77 82 10.1097/01.brs.0000193898.14803.8a 16395181

[B26] Petersen T Laslett M Thorsen H Manniche C Ekdahl C Jacobsen S Diagnostic classification of non-specific low back pain. A new system integrating patho-anatomic and clinical categories Physiotherapy Theory and Practice 2007 17 213 237 10.1080/716100585

[B27] SMS-Track Questionnaire 1.1.3 2007 New Agenda Solutions

[B28] Leboeuf-Yde C Axen I Jones JJ Rosenbaum A Lovgren PW Halasz L Larsen K The Nordic back pain subpopulation program: the long-term outcome pattern in patients with low back pain treated by chiropractors in Sweden J Manipulative Physiol Ther 2005 17 472 478 10.1016/j.jmpt.2005.07.003 16182020

[B29] Axen I Rosenbaum A Robech R Larsen K Leboeuf-Yde C The Nordic back pain subpopulation program: can patient reactions to the first chiropractic treatment predict early favorable treatment outcome in nonpersistent low back pain? J Manipulative Physiol Ther 2005 17 153 158 10.1016/j.jmpt.2005.02.007 15855901

[B30] Malmqvist S Leboeuf-Yde C Ahola T Andersson O Ekstrom K Pekkarinen H Turpeinen M Wedderkopp N The Nordic back pain subpopulation program: predicting outcome among chiropractic patients in Finland Chiropr Osteopat 2008 17 13 10.1186/1746-1340-16-13 18992154 PMC2588613

[B31] Landis JR Koch GG The measurement of observer agreement for categorical data Biometrics 1977 17 159 174 10.2307/2529310 843571

[B32] Kent PM Keating JL The epidemiology of low back pain in primary care Chiropr Osteopat 2005 17 13 10.1186/1746-1340-13-13 16045795 PMC1208926

